# A novel predictive model for new-onset atrial fibrillation in patients after isolated cardiac valve surgery

**DOI:** 10.3389/fcvm.2022.949259

**Published:** 2022-09-29

**Authors:** Heng Yang, Chen Yuan, Juesheng Yang, Haiyan Xiang, Wanqi Lan, Yanhua Tang

**Affiliations:** ^1^Department of Cardiovascular Surgery, The Second Affiliated Hospital of Nanchang University, Nanchang, China; ^2^The Second Clinical Medical College of Nanchang University, Nanchang, China

**Keywords:** postoperative atrial fibrillation, valvular heart surgery, perioperative, predictors, nomogram

## Abstract

**Background:**

Postoperative atrial fibrillation (POAF) is a severe complication after cardiac surgery and is associated with an increased risk of ischemic stroke and mortality. The main aim of this study was to identify the independent predictors associated with POAF after isolated valve operation and to develop a risk prediction model.

**Methods:**

This retrospective observational study involved patients without previous AF who underwent isolated valve surgery from November 2018 to October 2021. Patients were stratified into two groups according to the development of new-onset POAF. Baseline characteristics and perioperative data were collected from the two groups of patients. Univariate and multivariate logistic regression analyses were applied to identify independent risk factors for the occurrence of POAF, and the results of the multivariate analysis were used to create a predictive nomogram.

**Results:**

A total of 422 patients were included in the study, of which 163 (38.6%) developed POAF. The Multivariate logistic regression analysis indicated that cardiac function (odds ratio [OR] = 2.881, 95% confidence interval [CI] = 1.595–5.206; *P* < 0.001), Left atrial diameter index (OR = 1.071, 95%CI = 1.028–1.117; *P* = 0.001), Operative time (OR = 1.532, 95%CI = 1.095–2.141; *P* = 0.013), Neutrophil count (OR = 1.042, 95%CI = 1.006–1.08; *P* = 0.021) and the magnitude of fever (OR = 3.414, 95%CI = 2.454–4.751; *P* < 0.001) were independent predictors of POAF. The above Variables were incorporated, and a nomogram was successfully constructed with a C-index of 0.810. The area under the receiver operating characteristic curve was 0.817.

**Conclusion:**

Cardiac function, left atrial diameter index, operative time, neutrophil count, and fever were independent predictors of POAF in patients with isolated valve surgery. Establishing a nomogram model based on the above predictors helps predict the risk of POAF and may have potential clinical utility in preventive interventions.

## Introduction

Postoperative atrial fibrillation (POAF) represents the most common arrhythmic complication post-cardiac surgery and usually occurs within the first five postoperative days, with the peak incidence being on day two after surgery (1, 2). The occurrence of POAF reported in different studies ranges from roughly 10 to 60% (3–5), depending mainly on the type of surgery performed, with the highest rates undergoing valve or combined surgery (simultaneous coronary artery bypass graft surgery and valve surgery) (6). Despite advancements in surgical and anesthetic techniques, the incidence of POAF has not reduced significantly and (7, 8), in contrast, is expected to rise given an aging population. The large number of patients undergoing surgical valve surgery makes it essential to identify risk factors and establish effective management.

The development of POAF determines a significant increase in morbidity and mortality in post-cardiac patients (9). Although postoperative new-onset atrial fibrillation was once believed to be a self-limiting and benign complication, a growing body of evidence has suggested that POAF can result in a variety of serious adverse outcomes such as stroke, renal insufficiency, and acute cardiac failure (10–12), causing the increased length of intensive care unit and hospital stay (13, 14). Hence, it is very urgent to identify patients at high risk of POAF and to take preventive treatments during the perioperative period.

The mechanisms for developing new-onset AF after cardiac surgery are complex and not precisely known. Several factors have been identified as predictors for POAF based on previous studies, including advanced age, left atrium enlargement, left ventricular dysfunction, heart failure, and obesity (15–17). However, most of the above studies have focused on those patients after coronary artery bypass grafting (CABG). There are relatively limited data on risk factors or prediction model of POAF after isolated valve operations. This observational study aimed to identify independent predictors of POAF in patients following isolated heart valve surgery and to establish a convincing nomogram model for the early identification and timely management of POAF.

## Materials and methods

### Study population

This study was designed as a retrospective observational study. We examined the medical records of adult patients (18 years ≤ age ≤ 70 years) without previous AF who underwent isolated valvular surgery between November 2018 and October 2021 in our center. Patients were excluded if they met any of the following criteria: (1) with a previous history of atrial flutter, catheter ablation, or pacemaker installation; (2) Combined with severe coronary heart disease, infective endocarditis, congenital cardiac abnormalities, or cardiac tumors; (3) Combined with thyroid dysfunction; (4) complicated with serious comorbidities such as chronic vital organ failure, autoimmune diseases, malignant tumor or infection; (5) Combined with severe neurological or mental illness. All procedures for this study were carried out in compliance with the Helsinki Declaration. The protocol was approved by the Ethics Committee of the Second Affiliated Hospital of Nanchang University. The requirement of informed consent was waived because of the retrospective nature of this study and data anonymization was performed before analysis.

### Data collection

According to the development of new-onset POAF during the postoperative period until discharge, patients after heart valve surgery were stratified into POAF and non-POAF groups. Baseline characteristics and the clinical data of the two groups were collected, including demographics, comorbidities, preoperative variables, operative variables, and some postoperative variables. Missing data, including C-Reactive Protein (6.1%), erythrocyte sedimentation rate (5.4%), fibrinogen (2.3%), and glycosylated Hemoglobin (1.8%), were imputed using a multiple imputation model.

### Postoperative atrial fibrillation detection

Patients were transferred to the intensive care unit (ICU) at the end of the surgical operation, and continuous monitoring was available postoperatively using a cardiac rhythm monitor during ICU stay and the second or third postoperative day after discharge from the ICU. During inpatient ward follow-up, continuous electrocardiogram (ECG) monitoring was discontinued in clinically stable patients, and additional ECG recordings were obtained as soon as there was any suspicion of arrhythmia. POAF was defined as the occurrence of any episode of new-onset AF that lasted at least 30 s following surgery proved by routine ECG, ECG monitoring or course of disease records (10, 18).

### Definitions of important variables

Systolic blood pressure ≥ 140 mmHg, diastolic blood pressure ≥ 90 mmHg, or the usage of antihypertensive medication were all considered hypertension. Diabetes was defined as fasting serum glucose ≥ 7.0 mmol/L, random glucose ≥ 11.1 mmol/L, or the use of diabetic medication. Left atrial diameter index (LADi), left ventricular end-diastolic diameter index (LVDdi), and left ventricular end-systolic diameter index (LVDsi) were calculated by dividing left atrial diameter, left ventricular end-diastolic diameter, and left ventricular end-systolic diameter by body surface area (BSA), respectively. For the POAF group, postoperative data of patients were collected from the most recent recording before the onset of POAF, and data were collected on the third postoperative day for the non-POAF patients. The magnitude of fever was divided into four grades according to the maximal body temperature after the operation: no fever (37.3 < C), low fever (37.3°C–38.0°C), moderate fever (38.1°C–39.0°C), and high fever (> 39.0°C).

### Statistical analysis

Statistical analyses were performed by using SPSS26.0 and R (version 4.1.1). The measurement data was evaluated by the Kolmogorov-Smirnov test. The data were expressed as mean ± standard deviation if normally distributed, and an independent sample *t*-test or corrected *t*-test was performed. If the measurement data were non-normally distributed, it was expressed as median (first quartile-third quartile), and the Mann-Whitney *U* test was used for intergroup comparison. The chi-square test or Fisher’s exact test were used to compare categorical data presented as counts and percentages (%). Potential risk factors were identified using univariate analysis, and variables with statistically significant differences (*P* < 0.05) were further included in multivariate logistic regression analysis. The results of multivariate logistic regression analysis were expressed in odds ratio (OR) and 95% confidence interval (CI). A nomogram was constructed based on the multivariate analysis to predict the risk of POAF after isolated valve operation. The cohort was randomly divided into a training cohort and a validation cohort in a 7:3 ratio. The discrimination ability of the prediction model was assessed by C-index or the area under the receiver operating characteristic (ROC) curve (AUC). Nomogram model calibration was evaluated by the calibration curve. For internal validation, we applied the bootstrap method using 1000 replications. *P* < 0.05 is considered to be statistically significant.

## Results

### Epidemiology and patient characteristics

A total of 422 patients without preoperative atrial fibrillation who underwent isolated valve surgery were incorporated in the present study from November 2018 to October 2021. In total, 163 cases (38.6%) in 422 patients developed atrial fibrillation during postoperative hospitalization. POAF was most common on postoperative day 2 or 3 (Supplementary Figure 1) and tended to recur during hospitalization (Supplementary Figure 2). The median age of the patients in the POAF group was 57 years, compared with 53 years in the non-POAF group. 50.31% of patients who developed POAF were male, and 55.21% of patients in the non-POAF group. Details of the baseline characteristics and perioperative data of the two groups of populations were summarized in Tables 1, 2. For the POAF group, 25.15% of patients had hypertension, 5.52% had coronary artery atherosclerosis, and 5.52% had diabetes mellitus. In contrast, in the non-POAF group, hypertension was present in 21.24% of the patients, while 7.34% had coronary artery atherosclerosis, and 3.09 had diabetes.

**TABLE 1:** Baseline characteristics of patients with or without POAF.

Characteristics	POAF (*n* = 163)	Non-POAF (*n* = 259)	*P*-value
**Gender, n (%)**			0.325
Male	82 (50.31)	143 (55.21)	
Female	81 (49.69)	116 (44.79)	
Age, y	57 (49–63)	53 (46–61)	0.001
BMI, kg/m^2^	22.88 ± 3.26	22.61 ± 3.05	0.4
BSA, m^2^	1.58 ± 0.18	1.59 ± 0.16	0.550
**Blood pressure, mmHg**			
SBP	122 (111–134)	120 (110–136)	0.497
DBP	70 (64–76)	72 (65–80)	0.071
HR, BPM	79 (71–88)	79 (70–88)	0.863
**Cardiac function (NYHA), n (%)**			<0.001
II	18 (11.04)	90 (34.75)	
III	144 (88.34)	163 (62.93)	
IV	1 (0.61)	6 (2.32)	
Hypertension, n (%)	41 (25.15)	55 (21.24)	0.404
Coronary artery atherosclerosis, n (%)	9 (5.52)	19 (7.34)	0.55
Diabetes, n (%)	9 (5.52)	8 (3.09)	0.216
Smoker, n(%)	48 (29.45)	80 (30.89)	0.754
**Preoperative medications**			
β-Blocker, n (%)	33 (20.25)	68 (26.26)	0.159
ACE inhibitors/ARBs, n (%)	30 (18.4)	42 (16.22)	0.561
Calcium channel blocker, n (%)	28 (17.18)	32 (12.36)	0.167
Statins, n (%)	32 (19.63)	67 (25.87)	0.141
Diuretics, n (%)	64 (39.26)	110 (42.47)	0.515

Data are presented as n (%), mean ± standard deviation or median (first quartile-third quartile). BMI, body mass index; BSA, body surface area; BP, blood pressure; HR: Heart Rate; BPM, Beat Per Minute; NYHA, New York Heart Association.

**TABLE 2:** Perioperative factors of patients with or without POAF.

Variables	POAF (*n* = 163)	Non-POAF (*n* = 259)	*P*-value
**Preoperative factors**			
AAD, mm	33 (29–37)	32 (29–36)	0.238
LADi, mm/m^2^	27.66 (23.94–31.57)	25.108 (22.24–29.18)	<0.001
LVDsi, mm/m^2^	22.03 (18.64–25.53)	21.20 (18.86–25.42)	0.337
LVDdi, mm/m^2^	32.94 (28.73–36.60)	33.16 (29.35–36.84)	0.798
LVEF, %	61 (56–66)	63 (57–68)	0.035
CRP, mg/L	2.42 (1.58–4.81)	2.4 (1.51–5.19)	0.660
ESR, mm/h	17 (7–31)	15 (7–27)	0.185
Fibrinogen, g/L	2.45 (2.13–2.96)	2.37 (2.06–2.94)	0.165
HbA1c, %	5.6 (5.3–5.9)	5.4 (5.1–5.8)	0.002
STB, μmol/L	13.9 (11.46–18.09)	12.5 (9.76–17.32)	0.025
Cr, μmol/L	73.01 (60.48–91.71)	73.43 (62.12–84.27)	0.511
BUN, mmol/L	6.16 (4.6–7.35)	5.98 (4.66–7.24)	0.277
UA, μmol/L	355.84 (299.08–442.1)	360.3 (292.48–439.63)	0.739
K^+^, potassium ion, mmol/L	3.89 (3.66–4.15)	3.86 (3.66–4.14)	0.415
RBC count, × 10^12^/L	4.35 (4.04–4.7)	4.37 (4.02–4.73)	0.568
Platelet count, × 10^9^/L	188 (157–220)	191 (154–220)	0.991
Neutrophil count, × 10^9^/L	3.4 (2.69–4.36)	3.27 (2.58–4.15)	0.339
Lymphocyte counts, × 10^9^/L	1.62 (1.25–2.22)	1.61 (1.2–2.02)	0.29
Monocyte count, × 10^9^/L	0.38 (0.25–0.48)	0.36 (0.25–0.47)	0.597
Eosinophil count, × 10^9^/L	1.4 (0.32–2.7)	1.4 (0.16–2.5)	0.564
Hb, g/L	131 (120–141)	129 (120–140)	0.49
**Intraoperative factors**			
Aortic cross-clamp time, min	71 (55–93)	65 (53–84)	0.071
CPB time, min	102 (81–127)	93 (77–115)	0.042
Operative time, h	4.25 (3.75–5)	4 (3.5–4.5)	0.002
IntBT, n (%)	15 (9.20)	33 (12.74)	0.265
**Postoperative factors**			
STB, μmol/L	19.68 (15.14–27.66)	19.73 (15.02–25.78)	0.787
Cr, μmol/L	92.66 (72.76–126.5)	84.09 (66.71–104.21)	0.01
BUN, mmol/L	10.92 (9.24–14.3)	9.93 (7.83–12.37)	<0.001
UA, μmol/L	344.32 (236.4–500.33)	325.77 (216.54–438.39)	0.067
K^+^, mmol/L	4.1 (3.8–4.5)	4.1 (3.8–4.4)	0.955
Lactate, mmol/L	2 (1.3–2.9)	1.8 (1.4–2.5)	0.188
Blood glucose, mmol/L	9.4 (7.9–21.1)	9.3 (8.4–11.2)	0.751
RBC count, × 10^12^/L	3.36 (3.12–3.47)	3.46 (3.11–3.93)	0.201
Platelet count, × 10^9^/L	112 (90–139)	126 (96–153)	0.012
Neutrophil count, × 10^9^/L	12.45 (10.06–15.12)	10.45 (8.28–12.74)	<0.001
Lymphocyte counts, × 10^9^/L	1.19 (0.88–1.52)	1.13 (0.78–1.41)	0.138
Monocyte count, × 10^9^/L	0.69 (0.45–0.96)	0.76 (0.46–1.08)	0.267
Hb, g/L	99 (91–110)	101 (93–114)	0.09
Fever, n (%)			<0.001
No fever	4 (2.45)	44 (16.99)	
Low fever	38 (23.31)	137 (52.9)	
Moderate fever	98 (60.12)	61 (23.55)	
High fever	23 (14.11)	17 (6.56)	

Data are presented as n (%) or median (first quartile-third quartile). AAD, Maximal ascending aortic diameter; LADi, Left atrial diameter index; LVDsi, Left ventricular end-systolic diameter index; LVDdi, Left ventricular end-diastolic diameter index; LVEF, Left ventricular ejection fraction; CRP, C-reactive protein; ESR, Erythrocyte sedimentation rate; HbA1c, Glycated hemoglobin A1c; STB, Serum total bilirubin; Cr, Creatinine; BUN, Blood urea nitrogen; UA, Uric acid; K^+^, potassium ion; RBC, Red blood cell; Hb, Hemoglobin; CPB, Cardiopulmonary bypass; IntBT, Intraoperative blood transfusion.

### Independent risk factors for postoperative atrial fibrillation

Univariate analysis was performed on baseline characteristics (Table 1) and perioperative data (Table 2) of 422 patients to investigate potential risk factors for POAF. The results indicated that the following 13 factors were possibly associated with the risk of POAF occurring in patients after isolated valve surgery: age, cardiac function, left atrial diameter index, left ventricular ejection fraction, glycated hemoglobin, preoperative serum total bilirubin, cardiopulmonary bypass time, operative time, postoperative creatinine, postoperative blood urea nitrogen, postoperative platelet count, postoperative neutrophil count and magnitude of fever. Multivariable logistic regression was further conducted by adding the above factors and revealed that cardiac function (OR = 2.881, 95% [CI] = 1.595–5.206; *P* < 0.001), LADi (OR = 1.071, 95%CI = 1.028–1.117; *P* = 0.001), operative time (OR = 1.532, 95%CI = 1.095–2.141; *P* = 0.013), neutrophil count (OR = 1.042, 95%CI = 1.006–1.08; *P* = 0.021) and the magnitude of fever (OR = 3.414, 95%CI = 2.454–4.751; *P* < 0.001) were independent predictors associated with the occurrence of POAF in patients with isolated heart valve surgery (Table 3), while other features were not.

**TABLE 3:** Multivariate logistic regression analysis of screened variables.

Variables	Coefficient	SE	OR (95% CI)	*P*-value
Age	0.007	0.014	1.007 (0.981–1.034)	0.586
Cardiac function	1.058	0.302	2.881 (1.595–5.206)	<0.001
LADi	0.069	0.021	1.071 (1.028–1.117)	0.001
Pre-LVEF	−0.005	0.013	0.995 (0.97–1.02)	0.682
Pre-HbA1c	0.134	0.178	1.143 (0.806-1.621)	0.453
Pre-STB	0.023	0.015	1.023 (0.993–1.054)	0.13
CPB time	−0.005	0.005	0.995 (0.986–1.005)	0.313
Operative time	0.426	0.171	1.532 (1.095–2.141)	0.013
Post-Cr	0.002	0.003	1.002 (0.996–1.008)	0.449
Post-BUN	0.012	0.032	1.012 (0.95–1.078)	0.702
Post-platelet count	−0.002	0.003	0.998 (0.992–1.003)	0.428
Post-neutrophil count	0.041	0.018	1.042 (1.006–1.08)	0.021
Post-fever	1.228	0.169	3.414 (2.454–4.751)	<0.001

SE, Standard error; OR, odds ratio; CI, confidence interval; Pre-, Preoperative; Post-, Postoperative.

### Building a predictive model

**FIGURE 1:**
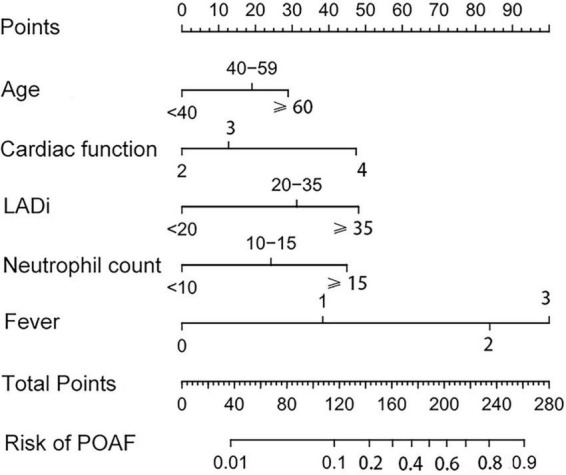
The nomogram for predicting new-onset atrial fibrillation following isolated cardiac valve surgery. LADi, left atrial diameter index.

**FIGURE 2:**
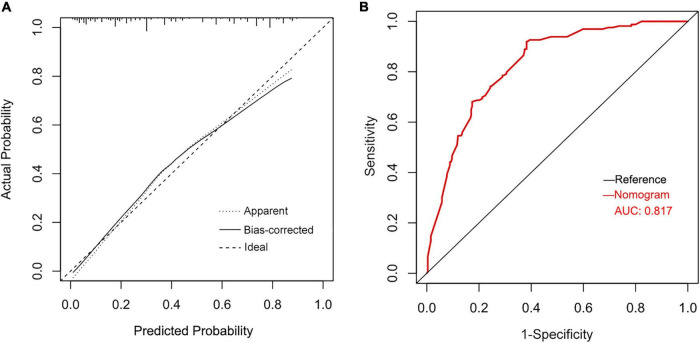
Calibration plots of the nomogram for predicting POAF after isolated valve surgery **(A)** and the ROC curve for the nomogram **(B)**.

Based on identified independent risk factors from multivariate logistic regression models, a nomogram was then constructed to predict the risk of POAF, and each variable was scored according to its regression coefficient (Figure 1). By calculating each point of the factors and then summing the points of all of them, the probability of POAF after valvular surgery in patients can be predicted. Internal validation was performed using 1,000 bootstrap resamples to test the predictive model’s performance. The C-index of the model was 0.810 by bootstrapping analysis. The calibration curve (Figure 2A) showed good agreement between predicted and actual outcomes. The ROC curve (Figure 2B) of the nomogram model for POAF was drawn, and the AUC was 0.817.

## Discussion

New-onset atrial fibrillation after cardiac surgery remains the most common complication with a reported incidence of approximately 30% (5, 19) and is growing in prevalence with the aging population. The reported incidence of POAF varied across studies, likely due to differences in diagnostic criteria, detection methods, and types of surgery among these studies (6). POAF occurs in 37 to 50% of patients undergoing valve-only surgery (4). In our present study, the occurrence of POAF was 38.6%, which was slightly lower than that reported in most previous studies, probably because patients over 70 were excluded from our research, while the increased age is commonly identified as a significant predictor of POAF (20, 21).

Postoperative atrial fibrillation recurrence is common and is associated with several adverse consequences, including stroke, renal failure, mortality, and increases the length of stay, risk of readmission, and resource usage (9, 11, 22, 23). Patients following CABG who developed POAF were at greater risk of cerebrovascular accidents in a *post hoc* analysis of 10-year outcomes (10). Moreover, Butt et al. (24, 25) demonstrated that POAF after valvular or non-cardiac surgery was associated with a similar risk of thromboembolism compared with non-surgical non-valvular atrial fibrillation, which confers a five-fold increased risk of ischemic stroke (26). Although many interventions have been made to reduce the occurrence of POAF (5, 27–31), the most effective management strategy remains uncertain. The present study results showed that cardiac function, LADi, operative time, neutrophil count, and the magnitude of fever were independent risk factors of POAF following isolated valve surgery, and then developed a predictive nomogram model by incorporating the above variables. This nomogram demonstrated a predictive performance with a good discriminative ability (C-index of 0.810) and calibration.

The left atrial enlargement has been observed in several previous studies associated with POAF (21, 32), which was also consistent with our study. A meta-analysis of patients who underwent cardiac surgery was conducted and showed that average preoperative left atrial volume indexed (LAVR) was higher in patients with POAF as compared to those without POAF (41.1 ml/m^2^ vs. 31.4 ml/m^2^, respectively) (33). Osranek et al. (34) also found that the patients with a left atrial volume (LAV) > 32 ml/m^2^ after heart surgery had a five-fold increased risk of POAF. Left atrial dilatation can lead to fibrosis of the atrial and adverse atrial remodeling, thereby altering the atrium’s electrical and mechanical properties, ultimately leading to multiple reentry pathways that induce atrial arrhythmias (7, 35). This study also found that preoperative cardiac function was associated with POAF, and patients with poor cardiac function were more likely to develop POAF, which agrees with a previous study (36, 37). Patients with impaired cardiac function may increase atrial load due to ventricular systolic and diastolic dysfunction, promoting atrial fibrosis (38, 39). In addition, cardiac structural abnormalities in patients with heart failure, such as reduced connexin, lead to susceptibility to AF. Left heart volume or pressure overload from impaired cardiac function leads to progressive left atrial dilation, deflecting conduction direction and shortening atrial refractory periods (39, 40).

The underlying mechanism of POAF is complex and thought to be caused by the combination of vulnerable substrates and triggers that promote POAF (41, 42). In other words, when there is atrial structural or electrical remodeling that makes them vulnerable to atrial fibrillation initiation, the presence of surgery-induced adverse factors such as ischemia, inflammation, oxidative stress, and autonomic imbalance will trigger POAF (43). We found that longer operative time was associated with a higher incidence of POAF, which is consistent with the study by Silva et al. (44), who suggested that an exceedingly long duration of surgery is a predictor of POAF. Understandably, the longer the operation, the more severe the risk factors such as ischemic injury or inflammation experienced by the patient (41). Notably, we did not find that the duration of cardiopulmonary bypass time and aortic cross-clamp time was significantly correlated with PAOF, which is varied in some previous studies (45, 46). Therefore, apart from the duration of cardiopulmonary bypass and aortic cross-clamp, the effects and underlying mechanisms of other possible factors in the duration of surgery on POAF, such as the use of anesthetics, deserve further study in the future.

Previous studies have proved that inflammation is one of the main underlying mechanisms of POAF (47). Patients who underwent cardiac operation with increased inflammatory levels, including an elevated white blood cell count and interleukin-6 levels, have a greater risk of atrial fibrillation (48, 49). In our study, a significant correlation was observed between the postoperative neutrophil count and the incidence of POAF. In addition, it has been found in some studies that neutrophils, myeloperoxidase activity, and Mitochondrial DNA in the pericardium of patients with POAF were elevated (50). Some factors, such as mitochondrial DNA, can activate the NLRP3 inflammasome in cardiomyocytes and then promotes AF (51). It has been reported (27) that posterior left pericardiotomy can significantly reduce the incidence of postoperative atrial fibrillation (17 vs. 32%), which also indicates that inflammatory factors in the pericardial fluid might play a vital role in POAF (52).

Our study found that postoperative body temperature, a factor rarely reported in previous similar studies, was significantly associated with POAF. Postoperative fever, especially non-infectious fever, is a common symptom after cardiac surgery, occurring in 60–70% of patients, caused mainly by surgical injury, cardiopulmonary bypass, and systemic inflammation (53). A reasonable explanation for our findings might be that fever can increase the heart rate, resulting in increased cardiac work and myocardial oxygen consumption, which may further aggravate myocardial damage and trigger POAF. Alternatively, it is possible that postoperative fever is simply a marker of the degree of damage or inflammation and does not directly affect the occurrence of POAF (54). The relationship and underlying mechanism between postoperative fever and POAF require further investigation. The magnitude of fever accounted for the highest score in our nomogram model, which indicates that timely intervention or early prevention of postoperative fever in patients who underwent surgery may be beneficial to reducing the occurrence of POAF. Multicenter prospective studies based on a large sample size should be designed to investigate whether this strategy is effective in reducing the events of POAF.

There are several limitations to the present study. Firstly, the nomogram model established was based on a single-center, retrospective study with a relatively small sample size and lacked external validation. Hence, the accuracy and generalizability of this predictive model still require validation through further studies. Secondly, because information about patients is primarily based on written records, some details of POAF, such as transient AF, may be overlooked due to limited documentation, which may lead to an underestimation of the incidence of POAF in our study. Thirdly, data on some clinical parameters were not included in our studies. Likewise, we did not record information on the occurrence of POAF after discharge.

## Conclusion

In summary, the incidence of POAF in patients underwent valve surgery was 38.6% in this study, and we identified five significant factors as predictors. We established a nomogram model for predicting POAF based on these factors and found that this model performed well in discrimination and calibration. The constructed model might help clinicians in decision-making by identifying high-risk populations after isolated valvular surgery and enabling guidance of proper prevention to reduce the occurrence of POAF.

## Data availability statement

The original contributions presented in this study are included in the article/Supplementary material, further inquiries can be directed to the corresponding author.

## Ethics statement

The studies involving human participants were reviewed and approved by the Ethics Committee of the Second Affiliated Hospital of Nanchang University. Written informed consent for participation was not required for this study in accordance with the national legislation and the institutional requirements.

## Author contributions

YT was responsible for the entire project and revised the draft. HY, CY, and WL performed the data extraction, statistical analysis, and interpreted the data. HY and JY drafted the first version of the manuscript. YT, JY, and HX revised the manuscript. All authors participated in the interpretation of the results and prepared the final version of the manuscript.

## Supplementary material

Supplementary Figure 1 Day of initial occurrence for postoperative atrial fibrillation in 163 patients in the POAF group.

Supplementary Figure 2 The number of postoperative atrial fibrillation occurrences during hospitalization in 163 patients in the POAF group.
