# Supplementary figures and images for: A novel predictive model for new-onset atrial fibrillation in patients after isolated cardiac valve surgery
Source: Front Cardiovasc Med. 2022 Sep 29;9:949259. doi: 10.3389/fcvm.2022.949259 (PMC9556269; doi:10.3389/fcvm.2022.949259)

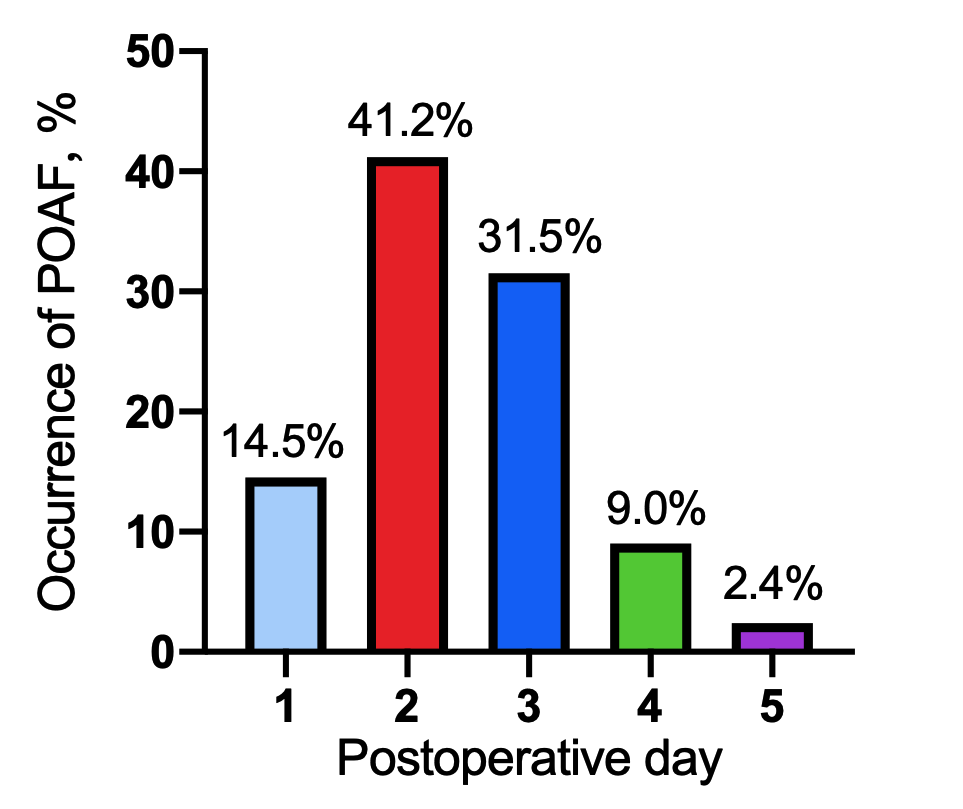

Supplement: Supplementary Figure 1 — Day of initial occurrence for postoperative atrial fibrillation in 163 patients in the POAF group. [file Image_1.TIF]

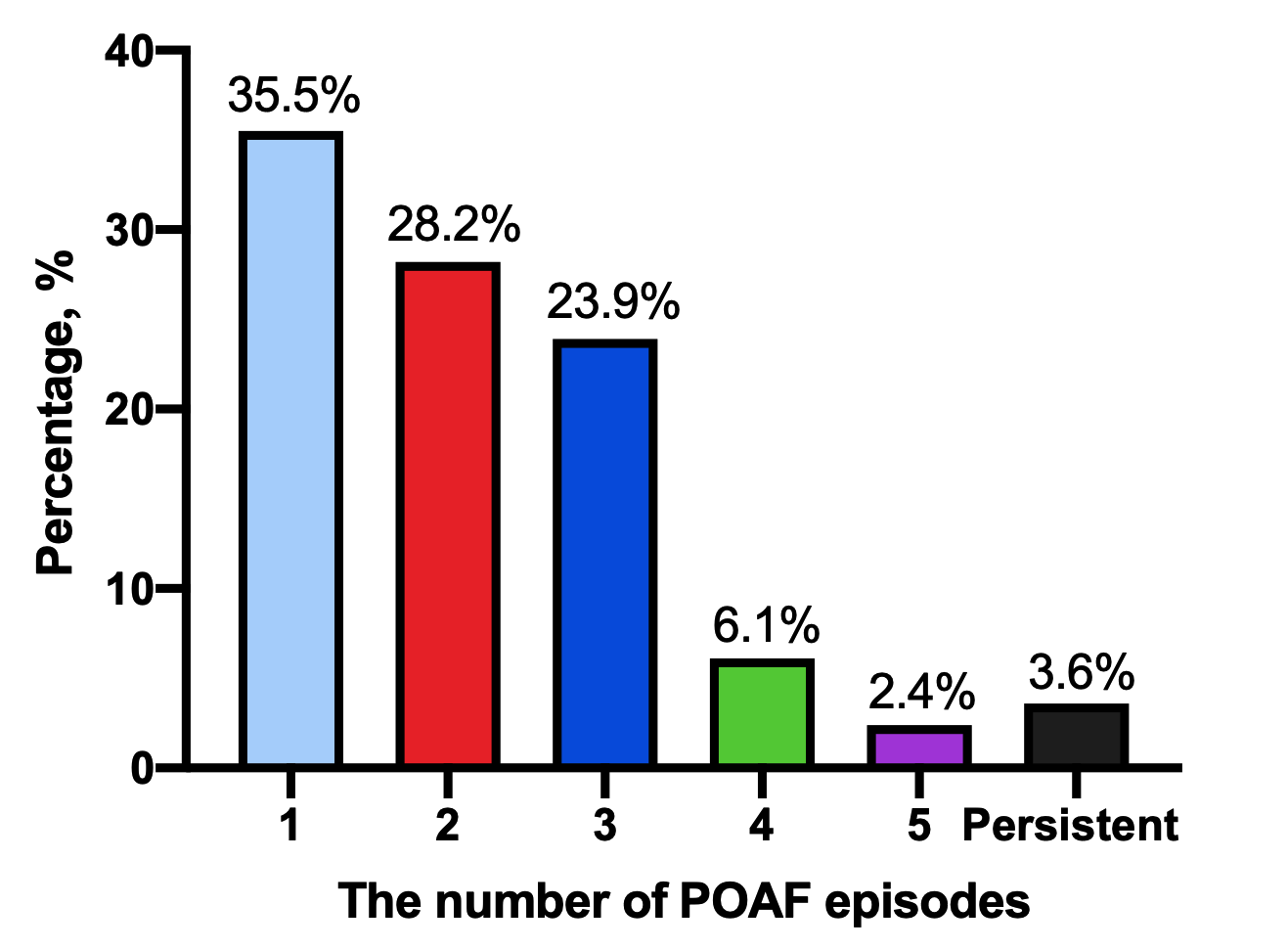

Supplement: Supplementary Figure 2 — The number of postoperative atrial fibrillation occurrences during hospitalization in 163 patients in the POAF group. [file Image_2.TIF]
